# Effectiveness of the Primary Trauma Care Course: Is the outcome satisfactory?

**DOI:** 10.12669/pjms.295.4002

**Published:** 2013

**Authors:** Masood Jawaid, Ayaz Ahmed Memon, Zubia Masood, Shams Nadeem Alam

**Affiliations:** 1 Dr. Masood Jawaid, MCPS, MRCS, FCPS, Assistant Professor Surgery, Dow University of Health Sciences, Karachi – Pakistan; 2 Dr. Ayaz Ahmed Memon, MCPS, MRCS, FCPS, Fellowship in Trauma and General Surgery; 3 Assistant Professor Surgery, Dow University of Health Sciences, Karachi – PakistanDr. Zubia Masood, FCPS, Assistant Professor Surgery, Hamdard College of Medicine and Dentistry, Karachi – Pakistan; 4 Prof. Shams Nadeem Alam, FRCS, Professor of Surgery, Dow University of Health Sciences, Karachi – Pakistan

**Keywords:** Primary trauma care, Trauma, effectiveness, evaluation, knowledge, skills

## Abstract

***Objective:*** To assess the improvement of knowledge and skills of trauma management among participant of Primary Trauma Care (PTC) workshop.

***Methods: ***A two days Primary Trauma Care (PTC) provider workshop was organized at Dow International Medical College, Karachi - Pakistan on March 5^th^ and 6th 2011. Participant’s knowledge was assessed by 30 Best Choice Questions (BCQs) and their trauma management skills were assessed by management of trauma case scenario both at pre and post workshop. All scenarios performed by participants were video recorded and marked on a 20 points check list and evaluated by two PTC trainers and graded after consensus. Percentage of participants who scored more than 70% marks on knowledge and skills component were also analyzed. Data was analyzed by SPSS version 17. Wilcoxon Sign Rank test was used to find out significant difference between pre and post workshop score.

***Results: ***A total of 20 participants attended the full workshop. Median One best question score before the workshop was 19.5 and post workshop was 25 (p<0.0001). Trauma skills scenario score also showed marked improvement with median score of 3.5 pre workshop and 9.5 post workshop (p<0.0001). Total 19 participants had ≥70% post workshop knowledge score. However, only 4 participants had trauma skill score with ≥70 marks.

***Conclusion: ***Primary Trauma Care workshop could be an effective course for gaining of knowledge and skills of initial management of trauma patients. However, some modifications need to be done for training of skills components to maximize the output of this interactive workshop.

## INTRODUCTION

Injuries due to trauma is a neglected epidemic in developing countries,^1^^,^^2^ accounting for more than five million deaths each year, approximately equal to the combined number of deaths from tuberculosis, malaria and HIV/AIDS. These facts represent only the tip of the 'injury pyramid', as: 'for every injury death there are 45 hospitals episodes, 630 doctor consultations and 5000–6000 minor injuries'.^3^

The American College of Surgeons developed the Advanced Trauma Life Support System (ATLS), which is now in practice in most developed western countries for treating severely injured trauma patients.^4^ However; it is a significant burden on the time and finances of trainees and leave burden for the trained instructors. Course fee is also high because of royalty fee payable to the American College of Surgeons. Apart from this, expensive diagnostic facilities like CT scanners, a standard protocol in ATLS and the large initial set-up costs make it unpractical in many third World countries.^5^ In reality; trauma management is drastically different in many developing countries. The reasons for this difference includes lack of resources, cost, trained human resource and trauma management education.^6^

The Primary Trauma Care (PTC) course was developed to teach doctors and other healthcare workers in the management of the severely injured patient at the District Hospital level, with both limited resources and manpower. PTC goal is to train people in the basics primary and secondary survey. The PTC program focuses on using minimal resources to maximum output. The first Primary Trauma Care course was started in Fiji and South Pacific (1997).^7^

In Pakistan, PTC courses were started in year of 2004 as joint venture between PTC Foundation, the IDEALS (International Disaster and Emergency Aid with Long Term Support) charity. Following the initial course, a PTC Board of Governors was established and large number of courses has been run at every corner of the country.^6^

There are numerous studies available about ATLS program worldwide^4^^,^^8^^,^^9^ but by extensive literature search we were able to retrieve only two studies about PTC.^6^^,^^10^ Out of them only one study from Iran evaluates the effectiveness of workshop and that too uses only MCQs for evaluation.^10^ To the best of our knowledge this is one of the first study which audits the effectiveness of the PTC workshop participants knowledge as well as skills of trauma management after two days of the provider workshop.

## METHODS

A two days Primary Trauma Care (PTC) provider workshop was organized at Dow International Medical College, Karachi - Pakistan on March 5^th^ and 6th 2011. Workshop was conducted in standard format proposed by Primary Trauma Care Foundation and comprises of lectures, practical skill stations and case scenarios on both days. The first day’s content included theoretical topics such as primary survey, secondary survey, airway management, chest trauma, shock and circulation evaluation; with skill practices on moulaged patient for airway and cervical spine management, in line neck immobilization, log roll, physical exam of trauma patient and chest tube placement on goat chest,. The second day’s content included theoretical topics such as trauma management of abdomen, head, spine, pediatric trauma, pregnant trauma patient, burns; with a practice on moulaged patient, neurological physical exam, trauma radiology, analgesia and patient transport. At the end of each day, they had case scenario practice based on the delivered lectures and skills stations. 

Participant’s knowledge was assessed by 30 Best Choice Questions (BCQs) pre and post as well and their trauma management skills were assessed by management of trauma scenario stations. A trauma scenario was used to evaluate the participants trauma management skills pre and post workshop. All participants performed scenarios were video recorded and marked on a 20 points check list. In addition, the degree to which the participants demonstrated an organized approach to trauma care (organized score) was also graded by the examiners, with scores varying from 1 to 5, the higher scores being superior to the lower scores. All video recording was evaluated by two PTC trainers and graded after consensus. Percentage of participants who scored more than 70% marks on knowledge and skills component were also analyzed.

Student’s feedback of the workshop was also assessed for the participant’s perceived value of the workshop. Data was analyzed by SPSS version 17. Non parametric test Wilcoxon Sign Rank test was used to find out significant difference between pre and post workshop score. 

**Table-I:** Median score (and IQR) before and after the PTC workshop

*Variable*	*Pre workshop* *Median (IQR)*	*Post workshop* *Median (IQR) *
One best Questions Score (0-30)	19.5 (16-22.5)	25 (23-27.7)
Trauma Scenario Skills Score (0-20)	3.5 (2.0-7.5)	9.5 (7.2-9.5)
Organized Score (0-5)	1.0 (1.0-2.0)	3.0 (2.0-3.75)

IQR = Inter quartile range (25% to 75%)

**Table-II:** Improvement in scores before and after the workshop of PTC (Statistical aspect

*Pairs*	*Z value*	*P value *
Pre and Post workshop BCQ score	-3.524	<0.0001
Pre and Post workshop Trauma Scenario Skills Score	-3.926	<0.0001
Pre and Post workshop Organized Score	-3.640	<0.0001

Wilcoxon Signed Ranks Test applied

**Table-III:** Feedback of students about the impact of the workshop

*Question*	*Strongly Agree* *(%)*	*Agree* *(%)*	*Undecided* *(%)*	*Disagree* *(%)*	*Strongly Disagree* *(%)*
Workshop objectives were clearly met	30	70	0	0	0
Enhance my knowledge and skills	65	35	0	0	0
Learned procedures correctly	40	50	5	5	0
Information presented is of practical value for me	70	25	5	0	0
Presentations were useful	35	65	0	0	0
Adequate time allowed for hands on practice	20	55	20	5	0
Questions were adequately responded	55	40	5	0	0

## RESULTS

A total of 21 doctors registered for the workshop. One participant was absent on second day so was excluded from the analysis. There were 12 females and 8 males. Eight participants were final year medical students, 8 interns and four participants were consultants.

Median One best question score before the workshop was 19.5 and post workshop was 25. Trauma skills scenario score and organized score also showed marked improvement with median score of 3.5 and 1.0 pre workshop and 9.5 and 3.0 post workshop respectively (Table – I). Wilcoxon Signed Ranks Test shows statistical significant difference between pre workshop and post workshop score in all domains of knowledge and skills (p<0.0001). (Table-II)

Total 19 participants had ≥70% post workshop knowledge score and 14 participants had post workshop score of ≥80%. However, only 4 participants had trauma skill score with ≥70 marks.

Participants feedback of the workshop showed that all agreed that objectives were met clearly and it enhanced their knowledge and skills. However, one fourth disagreed or was undecided that adequate time was allowed for hands on practice. (Table-III)

## DISCUSSION

The results showed that there is significant improvement of both participants’ knowledge and trauma management skills after two days of PTC workshop. All the participants rated the workshop highly informative and useful. The video recorded evaluation of trauma skills is one of the first evidence for the effectiveness of PTC workshop. A study from Iran reported significant improvement in knowledge after PTC workshop. In that study, the mean score in the pre-test was 18.84 and post-test was 26.72 (p<0.001), which is comparable to our study.^10^

It’s interesting to note that although there is marked improvement in knowledge of trauma management skills but only small number of participants scored more than 70% in skills. There can be multiple reasons for that. One is the participant’s interest, but overall all participants rated the workshop highly, they had paid a nominal fee (to cover the administrative costs) to attend the workshop and there was significant improvement in their trauma knowledge post workshop. Second reason may be facilitator’s factor. Out of 6 facilitators, three had been involved in conducting workshops overseas as well (i.e. China, Dubai, Jordan) and involved in training for more than a decade and facilitator were rated good by the participants. Probably, the reason behind low score was little one to one participant’s performance in scenarios and lack of personalized feedback. This was also evident by participant’s feedback that adequate time was not allowed for hands on practice (Table-III). To overcome this obstacle there is need to increase the demo scenarios with participant’s personal involvement. One option is to increase the duration of the workshop, which is not a very viable option as it is difficult for both the students and facilitator to take workshop leave from their busy clinical responsibilities. Second option is to increase the student to facilitator ratio and multiple individual scenarios performance with feedback. This model is like Mini CEX^11^, it will require intense involvement of both the student and facilitator in the workshop. We will be planning to conduct future courses at our own institute with this model and will document any improvement in the trauma management skills of the participants.

Another reason that why participant’s skills are important after PTC course is that PTC teaching is rapidly handed over to local instructors, thereby empowering local doctors and nurses early in the program,^6^ so its important for the future of PTC that its new instructors are trained appropriately.

Practical and effective PTC workshop are important specially in developing resource constrained countries as unlike other trauma training courses, the PTC Course is offered free to all countries wishing to use it. PTC Manual and Instructor packs are freely available on the web.^7^

We hope that these kinds of studies start generating interest in Primary Trauma Care foundation and the PTC instructors about importance of research aspect of PTC. There is need of more formal research not only about PTC provider workshop but also on participants long term retention of knowledge and skills their behavioral change in their own setup after the workshop, use of other approaches to be used in teaching and training like simulator, blended approach etc for more effectiveness of the workshop.

***Limitations of the study:*** Despite several limitations this study suffers from like small sample size, eight of the course participants being medical students who had very little clinical experience before they were exposed to this course hence not being ideal candidates for this course, these PTC workshops does provide some training and improvement in the skills of the participants which can improve the care of trauma patients at the Tehsil or District Hospital level. In many developing Third world countries with scarce financial and human resources, Primary Trauma Care courses could be a good beginning. However, it does not and cannot replace ATLS which remains the gold standard for primary trauma training for the physicians who are not dealing with injury as their primary practice. With little more improvements in contents, increase in case scenario management allowing more practical experience and careful selection of participants who have had enough clinical exposure, the Primary Trauma Care courses can prove to be quite useful and cost effective when resources and facilities does not permit advanced training like ATLS. 

## CONCLUSION

The two days Primary Trauma Care (PTC) workshop is a useful and informative course for the initial management of trauma patients. Workshop helps to increase the knowledge and skills of participants in different domains of trauma management. However some modifications need to be done for training of skills components to maximize the output of this interactive workshop.

## Notes

*Note: *All authors are instructors of PTC

*Source of Funding: *None
